# C’Est La Paix

**Published:** 1918-12

**Authors:** 


### WAR MEDICINE

Published by the American Red Cross Society in France
for the
Medical Officers of the American Expeditionary Forces

**EDITORIAL OFFICES:** **2, Place de Rivoli, Paris, Rooms 422-425**

WAR MEDICINE *accepts no original articles. Its scope is limited to the publication of the proceedings of the Research Society of the American Red Cross in France, abstracts of original articles, and editorial comment on subjects pertaining to the medicine, surgery, and hygiene of the war.*

*Circulars, bulletins, and reports from the Office of the Chief Surgeon of the American Expeditionary Forces will also appear in* WAR MEDICINE.

WAR MEDICINE *is distributed free of charge to the medical officers of the American Expeditionary Forces.*

*All communications pertaining to the mailing list should be addressed to the Editorial Offices. If copies are not received promptly and regularly the managing editor should be informed at once. Addresses should be written distinctly.*

THE RESEARCH SOCIETY OF THE AMERICAN RED CROSS IN FRANCE

*All communications should be addressed to the Secretary,*
*2, Place de Rivoli, Paris, Room 422*

### EDITORIAL COMMENTS

### C'EST LA PAIX

*La guerre finie* makes life to the medical officers of the American Expeditionary Forces a very different thing to what it was before the morning of the 11th of November. From the time that the American troops landed on French soil up to the day that the Armistice was declared, there were medical and surgical problems that worked to the limit of endurance every American physician and surgeon who has had the good fortune to be selected to come to France to fight in the battles against disease, and to care for our wounded soldiers. Until a few weeks ago few thought that the military machine which the Germans had been building up for the last half-century could be beaten into impotency before another year; but, when the cowardly Hun began to lose, and had to take a whipping every day wherever Marshal Foch cared to follow him and administer it, he slunk away and howled for mercy like a cur being lashed for a sheep-killing. All are glad that we shall have no more bloodshed, though there is some disappointment that the war ended before it had been carried into the heart of Germany and before Berlin had been captured by the Allied troops; but *c'est la paix* has come as a most agreeable surprise, and with it relaxation, and thoughts of the wonderful year through which we have passed. The present and the future also must be considered. It, therefore, seems appropriate to sum up the part that the American medical profession has played in the greatest crisis in the history of the world, and also to discuss the problems and opportunities for medical officers during the period when we are awaiting re-embarkation and demobilization, as well as to plan for the wonderful days when we shall enjoy again the delights and pleasures of work at home.

### THE HEROISM OF MEDICAL OFFICERS.

The men who went "over the top" cannot be praised too highly, but they deserve no more credit for the victory which has crowned and glorified Allied arms — and in which, by the way, every conscientious worker in our Armies has had a part in the making — than the medical officers who have fought the unseen enemies, — diseases, — that have wounded and killed more of our fighting men than have been slain, or have been placed on the non-effective list by German shot, shell and poison-gas. Neither have the fighting troops manifested finer courage than that shown by medical officers everywhere, — on the fighting line, in rendering first-aid to the wounded: in operating rooms, with Boche aeroplanes bombing the hospitals in which they were working; and in the pneumonia and meningitis wards of hospitals where, in administering to the needs of sick soldiers, they have daily and nightly risked their own lives. It requires as high a type of moral courage and as great physical bravery as that exhibited by soldiers, who face death at the cannon's mouth, for the medical officers to perform their duties at the first dressing stations and evacuation hospitals, and in the infectious wards of hospitals where they are constantly exposed to diseases that have caused more deaths than enemy shot and shell. The doctor who risks his life to save others is rewarded only by the consciousness of having performed his duty faithfully; while the soldier is decorated with honors and his deeds of valor are written in song and story. That the dangers which the doctors have faced in this war are real, is shown by the fact that the casualties in the Medical Corps are second only to those in the infantry.

### The Sacrifice for Service by Regular Medical Corps Surgeons.

Certainly in no other branch of the service in the United States Army have men made greater sacrifices to serve than have the physicians now with the Army in France. The military surgeons in the regular army, prior to the commencement of war, received their appointments by competitive examination; and medical college authorities advised only their best men to apply for commissions in the U. S. Army, Navy and Public Health Service, because they realized that the poorly prepared graduates in medicine, who had not received good hospital training, had no chance to become Army surgeons, particularly when there were many times more applicants for positions than there were vacancies to fill. The well prepared physicians who became medical officers in the Regular Medical Corps, in addition to foregoing the advantages of civil life, sacrificed opportunities in private practice which would surely have been several times more remunerative to them than the monetary reward they have received from their military service. The writer recalls two of the best prepared men in this class, both of whom, after graduation, served in the same hospital. One went into the Army and is now a Colonel, while the other entered civil practice, and, prior to the war, was earning an income running into six figures. The latter, however, left his home and his work and came to France to work with the French before the

United States entered the war. He has since been commissioned a Major in the Medical Corps, and has helped in a large way in the work of the Medical Department of the American Expeditionary Force. Recently he has been made a Colonel. This instance, which is but one of many parallel cases, is mentioned to show that the Regular Medical Corps officers have made many sacrifices in receiving the preparation for the administrative and executive positions which they have filled during the war.

### The Medical Reserve Corps.

When the history of the war has been written many of the brightest pages will be filled with the records of the American physicians and surgeons who gave up their homes and practices, which had required years of intensive effort to build up, to respond to the call of duty at the time when their country needed the best of her medical men. The medical profession of the United States stood the test of sacrifice for service to country and humanity, as well as that of efficiency in every line of medical and surgical work that was needed to help to win the war.

Even before the United States entered the conflict, many of our leading surgeons and many others of less reputation, but who were just as patriotic, came to France, England, Italy, Roumania, Serbia and Russia to give their services and risk their lives for the great cause; and when President Wilson declared war against Germany on April 6th, 1917, thousands of the best men in the medical profession offered their services to their country.

The fact that more than thirty thousand of the available estimated one hundred thousand men in active practice in the United States volunteered for service in our Army and Navy, showed that American doctors held duty and patriotism above dollars. Among these thirty thousand were the best in the profession, and there is no doubt that the private soldier in France has had the benefit of more skilful medical and surgical attention than can be obtained by the millionaire in any American city.

Of course every man who came into the medical service deserves praise for his self-sacrifice and patriotism, but the greatest credit belongs to those men who volunteered first, whether they were fortunate enough to be ordered to France or compelled to work in the United States. They not only have demonstrated their willingness to make sacrifices at a time when they were most needed, but they had the vision to see the opportunity for service.

It is a well known fact that many of these patriots have not received the recognition in rank that should have been accorded to them. In many cases they and their families have suffered because of inadequate salaries paid to medical officers; but it should be known that General Ireland has made every possible effort to rectify inequalities in rank, and that Chief Surgeon McCaw is still trying, with hope of success, to get through the promotions that these men so richly deserve.

The writer has heard many speak of this injustice in rank; but he has not heard one medical officer express any other sentiment than that of satisfaction at having served in France, and of the joy he has experienced in performing what he believed to be his simple duty. One Captain in the Medical Corps, who was a full " Colonel " in civil practice and a " Prince " in his home city, after mentioning the indignities he had endured from picking up cigarette stumps in a Medical Officers' Training Camp to being ordered about in hospitals by men twenty years his junior, said, " after all, the hardships and injustices, that seem to be a part of all branches of military service, do not count compared to the experiences that we have had, and the consciensciousness of knowing that we have stood God's test of manhood, by responding when our Country called ".

### The Record of the Medical Department of the A. E. F.

The statistics of the casualties and of the sick and death rates of our troops are being compiled, and the medical and surgical history of the American Expeditionary Forces is being written. Until this work is completed accurate information regarding the record of the Medical Department is not available. It is safe, however, using data that has been given out from time to time from the Chief Surgeon's Office, to predict that, considering the difficulties of transportation of men and supplies more than 3,000 miles from the United States to France, the conduct of the Medical Department of the American Expeditionary Forces has been such that we may feel justly proud of the record. Mistakes have been made, of course, but they have always been frankly admitted and corrected when they have been discovered, and the spirit of improvement and advancement has been manifested everywhere in every branch of medical service. The retrospect of the part that the American medical profession has had in winning the war, therefore, brings the satisfaction of the consciousness of "work well done"; and now that the horrors and hardships of war have passed, we should live up to the spirit of, and enjoy to the utmost, the victorious peace which medical officers with the American Expeditionary Forces have done their part to attain.

### THE PERIOD OF WATCHFUL WAITING BEFORE RE-EMBARKATION.

The emergency surgery of the war is over, but there is much work yet to be done before the responsibilites of the American medical profession in the war will have ended. The restoration of the sick and wounded, not for the battle front, but for the fight that each soldier must make for himself in the struggle for existence, and advancement in his work at home, must still go on; and the problems of preventive medicine are now paramount, because America needs for civil pursuits the brain and muscle of every soldier in France who has not made "the supreme sacrifice", and who can be spared from the Army of Occupation in Germany. Many medical officers will be needed in France for some time to come, for the prevention of disease must go on until all American troops have sailed for home.

Most of the physicians and surgeons from civil life desire to get back home a soon as possible, and Regular Army officers are anxious to return to their families, but the spirit of service and sacrifice that has been characteristic of American medical officers whether in the Regular Medical Corps or the Medical Reserve Corps is still dominant; and, with few exceptions, they express a perfect willingness to remain in France until there is no longer the need for their services with American troops.

Up to this time no definite policy of returning medical officers to the United States, and no plans for demobilization after their return have been decided upon — or at least none have been announced; but we may be assured that the Chief Surgeon and the Surgeon General are giving the matter careful consideration, and that they will handle the situation with fairness and justice to all. They wille xpedite re-embarkation and demobilization of the medical personnel of the army in every possible way. The transportation problem, however, for returning 2,000,000 men to America will be a difficult one to handle with satisfaction to the men, who feel that " the show is over " and that all that is left is to go home.

There is yet a great deal to do, and the return of American troops will be slower than our coming because the overcrowding of transports, when there was the urgent demand for fighting men in France, resulted in the spread of infections which could not be tolerated under other conditions. So we shall have to await our turn for orders to go home and we must accept the situation without complaint. The Biblical injunction "possess thy soul with patience " may well be practised by medical officers during the period of watchful waiting in France.

### Opportunities for Reading and Post-Graduate Work.

To philosophical medical officers, those who take advantage of opportunities for advancement during the days, weeks or months before the orders come to re-embark, the time may be spent profitably. Many doctors have been overworked during the recent drives, and most medical men in civil life had been overworked for years before entering the Medical Corps of the Army, so that a few weeks or months of rest and relaxation will prepare them for the hard work that will be necessary to rebuild their practices and to restore their finances to the status of pre-war days.

Many have been so busy with practice that for years they have not had time for keeping up with medical literature. Two or three hours of systematic reading of the medical journals (text books) that are in every hospital in the A. E. F. will result in the gaining of a lot of information that will be useful in private practice. There is a rumor that certain hospitals are planning courses in post-graduate instruction which will give admirable opportunities for those who are interested in their profession, and who desire to return to their work better doctors than when they came into the Army. It has also been reported that a certain proportion of medical officers who desire to visit the clinics in the cities of France and Great Britian will be allowed that privilege, provided of course that they can be spared from their regular duties. These are merely rumors, and if post-graduate instruction is carried on in the hospitals, or leaves allowed for attending French and British clinics, no doubt announcements will be made from the Chief Surgeon's Office.

### The Opportunity for Writing.

The families and friends of medical officers will want to know about their part in the war, and of their interesting experiences in France. An hour a day for a few weeks spent in writing a chronological record of the events through which a medical officer has passed will result in a kind of personal history covering his service in the greatest crisis of all time that would be of greater interest to his family and friends than any book on the war that has been or will be published. A few francs invested in a substantial memorandum book, and a little effort in writing are enough to prepare a memoir that will be treasured for generations to come by those who will always be proud that the hero of their family served in the greatest war in history.

The medical history of the war must be written, and every medical officer has the opportunity of being a historian, at least in so far as his work and his observations on any phase of medical and surgical matters are concerned. The historian of the Medical Department of the Army is anxious to receive the records of every man who may have observed anything of medical interest, and officers should consider it a duty to aid in the preparation of a medical history of the war that will go down through the ages as the best that has ever been written.

Medical Journals are anxious for articles on various phases of the hygiene, medicine and surgery of the war, particularly as they can be applied to practice in civil life. The period of watchful waiting will also give medical officers the opportunity of preparing papers for the medical journals back home who want to give their readers the benefit of what has been learned in the war. Then, too, many physicians will want to keep, for their own records, notes on the interesting cases that they have seen and other observations that will be helpful in their private practice, and which they are unwilling to trust to memory.

### The Duties of the Present.

Of course the first consideration of the medical officer will be the conscientious performance of his daily duties and the obligation not only to give the patients under his care the very best possible treatment, but to protect them and others from infections that lurk in hospital wards and in the barracks of soldiers. The time and the opportunity have come for educating the officers and soldiers of the line on personal and military (community) hygiene, not only to prevent disease and to assist in making a good record for the Medical Department of the American Expeditionary Force, but to prepare them to perform their part in the sanitation of the home communities when they return. Public health administration depends upon public opinion, and one of the compensations of war will be the education of several million you g men in the principles of sanitation; and when they return to their homes they will not tolerate the unsanitary conditions which existed and to which they paid no attention before they entered the Army.

The period of waiting orders to go back home may, therefore, be well spent by medical officers, and none of us should worry because we cannot return to our loved ones as soon as our hearts desire. While attending to our regular duties and in making good use of the leisure that some will have, we should enjoy to the fullest extent our sojourn in *la belle France*. Take care of the present; the future can wait. *C'est la paix.*

In the January number, editorial comment will be made on "The Medical Aftermath of the War."

